# Demographic and clinical characteristics of chikungunya patients from six Colombian cities, 2014–2015

**DOI:** 10.1080/22221751.2019.1678366

**Published:** 2019-10-21

**Authors:** Juan C. Rueda, Ana M. Santos, Jose-Ignacio Angarita, Rodrigo B. Giraldo, Eugenia-Lucia Saldarriaga, Jesús Giovanny Ballesteros Muñoz, Elías Forero, Hugo Valencia, Francisco Somoza, Daniel Martin-Arsanios, Elias-Josué Quintero, Viviana Reyes-Martinez, Diana Padilla, Francy M. Cuervo, Ingris Peláez-Ballestas, Mario H. Cardiel, Paula X. Pavía, John Londono

**Affiliations:** a Biosciences Doctoral Programme, Faculty of Medicine and Engineering, Universidad de La Sabana, Chía, Colombia; b Grupo Espondiloartropatías, Department of Rheumatology, Universidad de La Sabana, Chía, Colombia; c Department of Rheumatology, Hospital Militar Central, Bogotá, Colombia; d Department of Rheumatology and Internal Medicine, Universidad del Norte, Barranquilla, Colombia; e Rheumatology Unit, Hospital General de México “Doctor Eduardo Liceaga”, Mexico City, Mexico; f Centro de Investigación Clínica de Morelia SC, Morelia, Mexico; g Unidad de Investigación Científica, Hospital Militar Central, Bogotá, Colombia

**Keywords:** Chikungunya virus, Colombia, Epidemic, South America, Arbovirus

## Abstract

In 2014, the chikungunya virus reached Colombia for the first time, resulting in a nationwide epidemic. The objective of this study was to describe the demographics and clinical characteristics of suspected chikungunya cases. Chikungunya infection was confirmed by enzyme-linked immunosorbent assay and 548 patients where included in the study. Of these patients, 295 were positive for antibodies against chikungunya (53.8%), and 27.6% (151/295) were symptomatic for chikungunya infection, with a symptomatic:asymptomatic ratio of 1.04:1. Factors associated with infection included low income and low socio-economic strata (odds ratio [OR]: 1.8; 95% confidence interval [CI]: 1.0–3.2, *p* = 0.003 and OR: 2.1; CI: 1.3–3.4, *p* = 0.002, respectively). Confirmed symptomatic cases were associated with symmetric arthritis (OR: 11.7; CI: 6.0–23.0, *p* < 0.001) of ankles (OR: 8.5; CI: 3.5–20.9, *p* < 0.001), hands (OR: 8.5; CI: 3.5–20.9, *p* < 0.001), feet (OR: 6.5; CI: 2.8–15.3, *p* < 0.001), and wrists (OR: 17.3; CI: 2.3–130.5, *p* < 0.001). Our study showed that poverty is associated with chikungunya infection. Public health strategies to prevent and control chikungunya should focus on poorer communities that are more vulnerable to infection. The rate of asymptomatic infections among confirmed cases was 48.8%. However, those with symptoms displayed a characteristic rheumatic clinical picture, which could help differentiate chikungunya infection from other endemic viral diseases.

## Introduction

Chikungunya virus (CHIKV) is an alphavirus from the *Togaviridae* family that causes acute arthropathy in humans and other animals [1–3]. Transmission usually starts with an infected mosquito bite after which the virus infects fibroblasts and macrophages in the dermis [4]. After an incubation period of 3–7 days, it is disseminated through the lymphatic system and bloodstream to epithelial and endothelial cells, and other tissues and cells [3,4]. The virus replicates causing viraemia, fever, rash, myalgia, arthralgia, and arthritis [5]. At this point, the acute phase is established, lasting for approximately 2 weeks and characterized by the appearance of immunoglobulin type M (IgM) (persisting for up to 3 months) followed by the production of immunoglobulin type G (IgG), which provides antiviral immunity for years [5,6]. After the acute phase, CHIKV infection can progress to a chronic stage where rheumatic symptoms can last for several months to years [5,7]. Indeed, studies have found high frequencies of persistent joint pain after 32 months of CHIKV infection and even as high as 59% after 6 years, with patients fulfilling criteria for rheumatoid arthritis, spondyloarthritis, and undifferentiated polyarthritis, posing a diagnostic challenge to the primary care physician and the rheumatologist [8–10]. A recent study in our country demonstrated persistent relapsing-remitting joint pain in 1 out of 8 patients with serologically confirmed CHIKV infection after 3 years [11].

In 2014, the Colombian Rheumatology Association started the task of establishing the prevalence of rheumatic diseases in the country. The strategy used to identify rheumatic diseases was the Community Oriented Program for Control of Rheumatic Diseases (COPCORD), which has proven effective in other Latin American countries [12–15]. COPCORD is a low-budget, community-oriented programme to measure and evaluate pain and disability from rheumatic disorders in developing countries [12,16].

During the initial phase of the COPCORD study, a CHIKV epidemic struck Colombia from August 2014 to September 2015 [17,18]. Because the main complaint in CHIKV is musculoskeletal (MSK) symptoms, the number of cases identified by the COPCORD study increased. Therefore, CHIKV-infected patients had to be distinguished within the studied population.

In August 2014, CHIKV first arrived in northern Colombia, causing 106.763 reported cases in the first year and spanning the whole territory (32 state departments) with *Aedes (Ae.) aegypti* as the only vector, since the Asian lineage is the only genotype described up to date in our country [17,19–25]. Specifically, the first autochthonous cases of CHIKV infection notified to the Colombian Health Ministry were from the municipality of Mahates, a town located in the Bolivar department; a territory in the Caribbean region, limiting with the north-western Caribbean sea (Atlantic Ocean) of Colombia [17]. According to the Pan-American Health Organization (PAHO) statistics, Colombia was in third place of cumulative cases in the Americas, with 294,831 cases, following the Dominican Republic with 539,362, and Brazil with 773,010 cases [26]. By the end of 2015, the Colombian Health Ministry declared the end of the epidemic; however, cases have continued to be reported up to now, with reports of 346 notified cases at epidemiological week 28 of 2019 in Colombia (312 clinically confirmed, 6 laboratory confirmed, and 28 suspected cases) [18,27–29].

This study investigated individuals with rheumatic symptoms and suspicion of CHIKV infection from the Colombian COPCORD cohort during 2014 and 2015. Our objective was to evaluate patients’ clinical presentation, as well as demographic and socioeconomic characteristics.

## Materials and Methods

### Study population

This was a cross-sectional analysis nested in a community cohort, including patients aged >18 years. The COPCORD uses a stratified sampling method in three stages. The first sampling stage consisted of selecting cartographic areas in each city, as defined by the Colombian Statistics Administration Department (DANE, Departamento Administrativo Nacional de Estadística). The second stage involved blocking each sector using an urban analysis tool that classifies cities into blocks, houses, households, and people (VIHOPE). The third stage concerned the homes in each block; all household members were surveyed. The sample size was calculated at 6528 individuals for a 1.5 sampling design effect and 14% sampling error [30].

The COPCORD questionnaire adapted for Colombia was used by trained interviewers between August 2014 and September 2015 at each individual’s house [31,32]. Through this questionnaire, information on demographic and socioeconomic variables was gathered. These included monthly income and a socioeconomic stratification system used in Colombia. In a pilot COPCORD study, most patients refused to provide an exact amount of monthly income. To improve the response rate to that question, aleatory ranges were established as shown in Table 1. In Colombia, residential buildings that must receive public services are classified in a socioeconomic system ranging from 1 to 6, with 1 as the lowest income area and 6 as the highest. This stratification is carried out mainly to assign subsidies and collect contributions differentially by strata. Thus, those with more economic capacity (5 and 6) pay more for public services so that the lower strata (1–3) can pay their bills [33]. These six socioeconomic strata classify households based on the type of construction, number of habitable rooms, water and sanitation conditions (presence of indoor toilet, existence of piped water supply, etc.), and level of urbanization of each home’s location (paved roads, availability of sewage system, etc.) [33,34].

**Table 1.** Demographics in patients with suspected CHIKV infection.

	IgG or IgM for CHIKV			
	*Positive*^a^** **(*n* = 295)	*Negative*^b^** **(*n* = 253)	OR (CI)	*p*-value
Age in years (mean ± SD)	49.6 ± 17.4	48.9 ± 17.6		
Female	208 (54.5%)	174 (45.5%)		
*Race*				
Mestizo (*n*: 292)	145 (49.7%)	147 (50.3%)		
Caucasian (*n*: 188)	110 (58.5%)	78 (41.5%)		
Afro-American (*n*: 41)	29 (70.7%)	12 (29.3%)	2.19 (1.09–4.38)	0.024
Amerindian (*n*: 24)	8 (33.3%)	16 (66.7%)	0.41 (0.17–0.98)	0.039
*Origin*				
Barranquilla (*n*: 248)	165 (66.5%)	83 (33.5%)	2.60 (1.83–3.68)	<0.001
Bogotá (*n*: 90)	45 (50.0%)	45 (50.0%)		
Medellín (*n*: 84)	18 (21.4%)	66 (78.6%)	0.18 (0.10–0.32)	<0.001
Cúcuta (*n*: 55)	36 (65.5%)	19 (34.5%)		
Bucaramanga (*n*: 47)	14 (29.8%)	33 (70.2%)	0.33 (0.17–0.63)	0.001
Cali (*n*: 24)	17 (70.8%)	7 (29.2%)		
*Monthly income*				
None (*n*: 199)	107 (53.8%)	92 (46.2%)		
<157 USD (*n*: 90)	62 (68.9%)	28 (31.1%)	2.13 (1.32–3.46)	0.002
158–315 USD (*n*: 189)	95 (50.3%)	94 (49.7)		
316–471 USD (*n*: 41)	16 (39.0%)	25 (61.0%)		
472–630 USD (*n*: 15)	9 (60.0%)	6 (40.0%)		
631–725 USD (*n*: 3)	2 (66.7%)	1 (33.3%)		
726–906 USD (*n*: 7)	3 (42.9%)	4 (57.1%)		
>906 USD (*n*: 4)	1 (25.0%)	3 (75.0%)		
*Socioeconomic strata*				
Stratum 1 (*n*: 169)	115 (68.0%)	54 (32.0%)	2.35 (1.60–3.44)	<0.001
Stratum 2 (*n*: 226)	104 (46.0%)	122 (54.0%)	0.58 (0.41–0.82)	0.002
Stratum 3 (*n*: 114)	60 (52.6%)	54 (47.4%)		
Stratum 4 (*n*: 32)	9 (28.1%)	23 (71.9%)	0.31 (0.14–0.69)	0.003
Stratum 5 (*n*: 7)	6 (85.7%)	1 (14.3%)		

CHIKV: chikungunya virus; IgM: immunoglobulin M; IgG: immunoglobulin G; OR: odds ratio; CI: 95% confidence interval; SD: standard deviation, USD: United States dollar.

^a^fulfilled World Health Organization Criteria for confirmed case of CHIKV; ^b^did not fulfil World Health Organization Criteria for confirmed case of CHIKV.

Individuals were considered COPCORD-positive patients if they reported non-traumatic MSK symptoms including pain, stiffness, or arthritis during the 7 days before the interview. Because the chikungunya epidemic reached Colombia during the study, interviewers included chikungunya-related symptoms such as fever, rash, myalgia, and fatigue and possible CHIKV direct diagnosis in their questionnaire. The suspicion of CHIKV infection was based on patients’ reports.

If a patient was positive for COPCORD and CHIKV infection was suspected, a follow-up examination was conducted in the next 7 days by a trained rheumatologist or a rheumatology fellow, during which chikungunya fever was confirmed according to World Health Organization (WHO) criteria. Then, a specific questionnaire was administered including time of disease onset and further symptoms, such as joint, dermatological and gastrointestinal manifestations [35]. Blood samples were also taken. Patients were evaluated only once and were excluded if the examiner suspected or confirmed a rheumatic disease. The definitions of arthralgia and arthritis used were taken from Woolf [36].

### Case definitions for CHIKV infection according to WHO criteria [35]

A case was considered suspect based on clinical criteria (acute onset of fever >38.5°C and incapacitating joint pain) and epidemiological criteria (residing in or having visited areas that had reported transmission within 15 days prior to the onset of symptoms). A case was confirmed when the patient met laboratory criteria irrespective of clinical presentation (presence of virus-specific IgM or IgG antibodies in a single serum sample collected in the acute or convalescent stage, respectively). Because our population was immunologically naïve (there were no reports of CHIKV infection prior to this epidemic) we considered as positive the presence of virus-specific IgG antibodies in single serum sample during any stage of the disease.

### CHIKV serology

Enzyme-linked immunosorbent assay (ELISA) was performed according to the manufacturer’s instructions (Abcam® ab177848 anti-CHIKV IgM human ELISA kit and ab177835 anti-CHIKV IgG human ELISA kit, Abcam, Cambridge, UK). Abcam’s anti-CHIKV IgM Human ELISA kit is reported to produce comparable results to the Centers for Disease Control and Prevention (CDC) IgM ELISA [37,38]. Analytical specifications according to manufacturer state a specificity >90% and sensitivity >90% for both IgM and IgG anti-CHIKV.

No cross-reactivity against *Bordetella pertussis*, *Chlamydia trachomatis*, *Chlamydia pneumoniae*, dengue virus, tick-borne encephalitis (TBE), *Helicobacter pylori*, herpes simplex virus 2, *Leishmania*, *Mycoplasma,* or *Schistosoma* has been reported for IgM. No cross-reactivity against dengue virus, TBE, cytomegalovirus, Epstein–Barr virus, or *Helicobacter pylori* has been reported for IgG. The manufacturer reports a 10% rate of misclassification with IgG serology. In our cohort of positive IgG patients with possible CHIKV infection, 10% represents about 5 patients, which is not statistically relevant and therefore does not affect our results.

### Statistical analysis

Descriptive analysis was made using means and standard deviation (SD) for continuous variables and count and percentages for categorical variables. Two by two tables were used to establish associations between categorical variables. Odds ratios (OR) were calculated for associations with 95% confidence intervals (CI), and Student’s *t*-test used to compare means, regarding *p* < 5% as statistically significant. SPSS version 22.0 (IBM, Armonk, NY, USA) was used for data analysis.

### Ethical considerations

This study was carried out according to the Declaration of Helsinki 2013. Informed consent was obtained, prior to the patients’ admission. The study was approved by the ethics committee from La Universidad de La Sabana (study approval MED-197-2015) and the Hospital Militar Central (study approval 106-2016).

## Results

**Figure 1.**
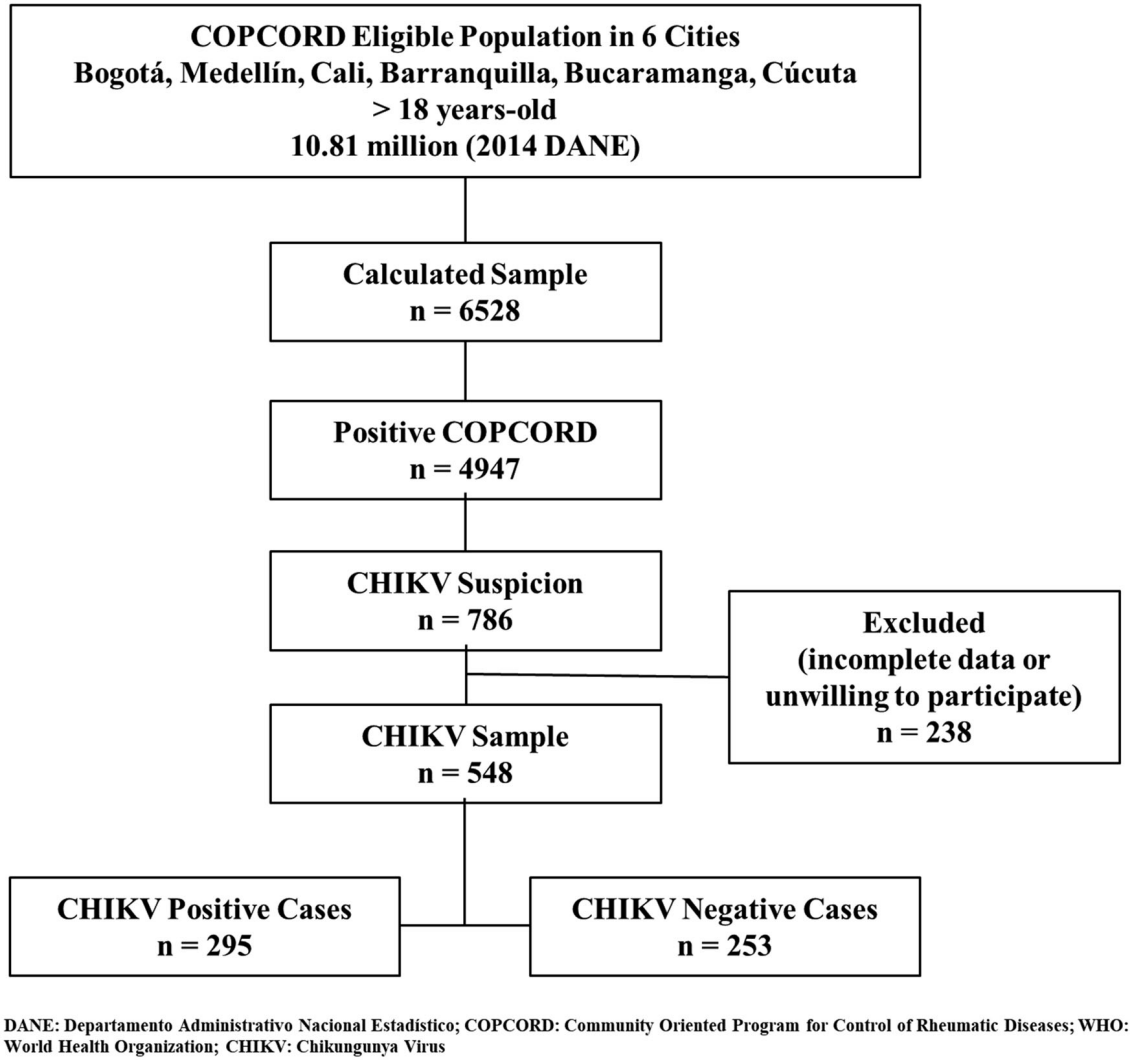
Profile of the study population. WHO: World Health Organization; CHIKV: chikungunya virus.

Of the 6528 people surveyed in the COPCORD study, 548 (8.4%) were included in our study as suspected for CHIKV infection. From this subgroup, only 177 (32.3%) fulfilled the WHO criteria for suspected CHIKV infection; however, 295 (53.8%) were positive for CHIKV IgG or IgM, and thus fulfilled the WHO criteria for confirmed CHIKV infections. Among these confirmed cases, 151 (51.2%) reported CHIKV symptoms (Figure 1). The following analysis was made between patients with positive CHIKV infection (confirmed cases) and negative CHIKV infection (*n*: 253, 46.2%). Positivity of IgM was found in 6.8% (*n*: 20) patients, concomitant positivity of IgM and IgG in 21.3% (*n*: 63), and positivity of IgG in 71.9% (*n*: 212).

### Demographics

For patients with positive CHIKV serology, 57.6% (*n* = 170) were >45 years of age. Of all 548 studied patients, most were female (*n* = 382, 69.7%); however, there was no difference between confirmed CHIKV-infected patients and uninfected patients (Table 1). Mestizos (*n* = 292; 53.3%) were the most common race in the studied population, and being Afro-American was associated with CHIKV infection (*p* = 0.024; OR: 2.19, CI: 1.09–4.38).

**Figure 2.**
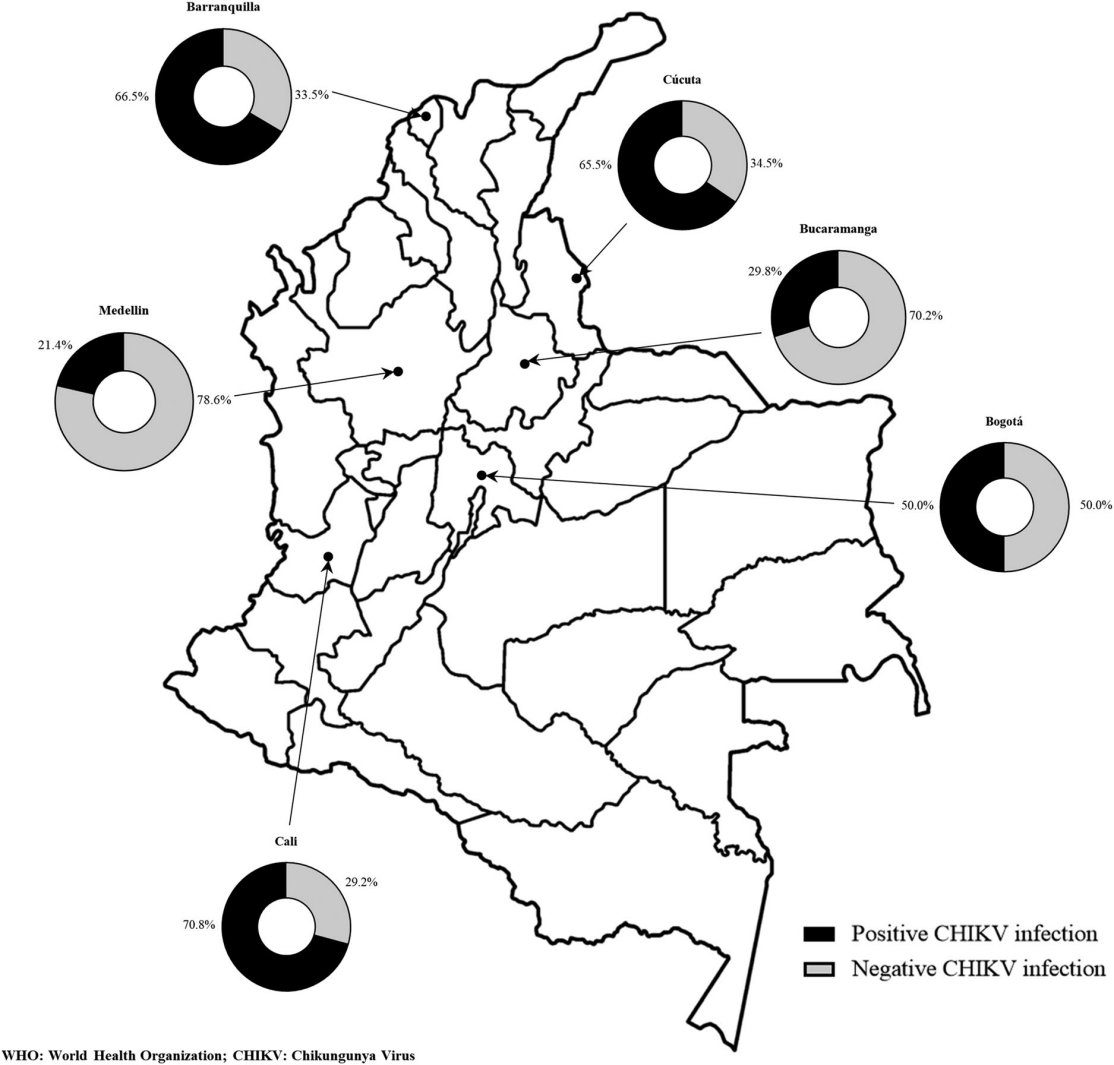
Distribution by city according to WHO criteria and CHIKV serology. DANE: Departamento Administrativo Nacional de Estadística; COPCORD: Community Oriented Program for Control of Rheumatic Diseases; WHO: World Health Organization; CHIKV: chikungunya virus.

Barranquilla was the city with most confirmed CHIKV-infected patients in general (*n* = 165; 55.9.%) conferring an association with CHIKV infection of 2.6 times (*p* < 0.001; OR: 2.6, CI: 1.8–3.6) (Table 1). Interestingly, Bogotá, a city with no *Ae. aegypti* due to its altitude (2630 m above sea level), had the second most confirmed CHIKV-infected patients (*n*: 45, 15.3%) (Figure 2).

Of all 548 patients, 92.7% (*n*: 508) had some type of education. Primary school education was the most frequent in confirmed cases (*n* = 87, 29.5%). Of those, 93.9% (*n* = 277) had knowledge of how to read and write. Of interest, basic primary school education was associated with not having CHIKV infection (*p* = 0.048; OR: 1.43, CI: 1.00–2.05).

Of confirmed CHIKV-infected patients, 107 (36.3%) had no monthly income. Most patients with an income below 157 USD per month (*n* = 62, 68.9%) had confirmed CHIKV infection (*p* = 0.002; OR: 2.13, CI: 1.32–3.46) (Table 1). Confirmed CHIKV infection was associated with socioeconomic stratum 1 (*n*: 115, 68%; *p* < 0.001, OR: 2.35, CI: 1.60–3.44). More than half of confirmed patients (*n* = 134, 59.0%) were housewives, which was associated with CHIKV infection (*p* = 0.04; OR: 1.43, CI: 1.01–2.01).

### Clinical features

#### Joint involvement

Ninety-one per cent of patients with confirmed CHIKV infection had arthralgia (*n* = 270), and most of these (88.8%) were symmetric (*n* = 240). However, only 33.6% had arthritis (*n* = 99), of whom 90 (90.9%) had the symmetric pattern. The most frequent painful joints were knees (*n* = 184, 62.4%), hands (*n* = 158, 53.6%), ankles (*n* = 137, 46.4%), feet (*n* = 104, 35.3%), and wrists (*n* = 93, 31.5%).

**Figure 3.**
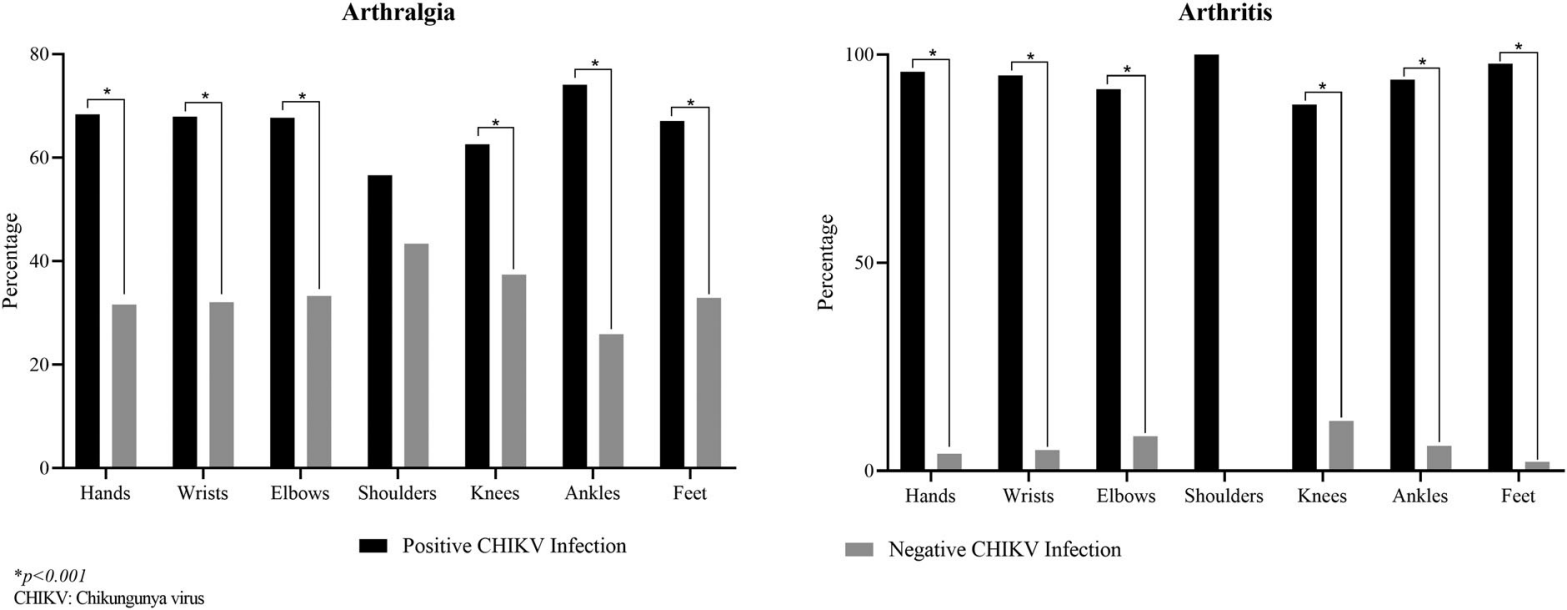
Joint involvement in confirmed CHIKV-infected patients. *p < 0.001.

In general, arthritis was less common (Table 2). Hands and ankles were the most affected joints (*n* = 47, 15.9% each), followed by feet (*n* = 45, 15.3%), knees (*n* = 22, 7.5%), and wrists (*n*: 19, 6.4%). Overall, confirmed CHIKV-infected patients were more affected compared to CHIKV-negative patients. The difference was more evident with joint inflammatory involvement of feet, hands, wrists, and ankles (Figure 3 and Table 2).

**Table 2.** Joint involvement in patients with suspected CHIKV infection.

	IgG or IgM for CHIKV		** **	
	*Positive*^a^** **(*n* = 295)	*Negative*^b^** **(*n* = 253)	OR (CI)	*p-*value
*Arthralgia*				
Symmetric (*n*: 382)	240 (62.8%)	142 (37.2%)	3.41 (2.32–5.00)	<0.001
Hands (*n*: 231)	158 (68.4%)	73 (31.6)	2.84 (1.99–4.05)	<0.001
Wrists (*n*: 137)	93 (67.9%)	44 (32.1%)	2.18 (1.45–3.28)	<0.001
Elbows (*n*: 111)	74 (66.7%)	37 (33.3%)	1.95 (1.26–3.02)	0.002
Shoulders (*n*: 143)	81 (56.6%)	62 (43.4%)		
Knees (*n*: 294)	184 (62.6%)	110 (37.4%)	2.15 (1.53–3.03)	<0.001
Ankles (*n*: 185)	137 (74.1%)	48 (25.9%)	3.70 (2.51–5.46)	<0.001
Feet (*n*: 155)	104 (67.1%)	51 (32.9%)	2.15 (1.46–3.18)	<0.001
*Arthritis*				
Symmetric (*n*: 96)	90 (93.8%)	6 (6.3%)	18.07 (7.74–42.15)	<0.001
Hands (*n*: 49)	47 (95.9%)	2 (4.1%)	23.78 (5.71–98.98)	<0.001
Wrists (*n*: 20)	19 (95.0%)	1 (5.0%)	17.34 (2.30–130.52)	<0.001
Elbows (*n*: 12)	11 (91.7%)	1 (8.3%)	9.76 (1.25–76.13)	0.008
Shoulders (*n*: 9)	9 (100.0%)	0 (0.0%)		
Knees (*n*: 25)	22 (88.0%)	3 (12.0%)	6.71 (1.98–22.71)	<0.001
Ankles (*n*: 50)	47 (94.0%)	3 (6.0%)	15.79 (4.85–51.41)	<0.001
Feet (*n*: 46)	45 (97.8%)	1 (2.2%)	45.36 (6.20–331.61)	<0.001

CHIKV: chikungunya virus; IgM: immunoglobulin M; IgG: immunoglobulin G; OR: odds ratio; CI: 95% confidence interval.

^a^fulfilled World Health Organization criteria for confirmed case of CHIKV; ^b^did not fulfil World Health Organization criteria for confirmed case of CHIKV.

#### Systemic features

Fever was present in only half of the patients with confirmed CHIKV infection (*n* = 151, 51.2%; *p* < 0.001, OR: 9.15, CI: 5.74–14.58), as was myalgia (*n* = 139, 47.1%; *p* < 0.001, OR: 8.91, CI: 5.48–14.48), which was mostly in the extremities (*n* = 96, 32.5%). Also, fatigue was reported by patients in 58.6% of patients (*n* = 173) with confirmed infection (*p* < 0.001, OR: 10.54, CI: 6.74–16.46).

#### Dermatological involvement

The pruritic maculopapular rash was present in 44.7% (*n* = 132) of confirmed CHIKV-infected patients (*p* < 0.001, OR: 9.97, CI: 5.92–16.79). Overall, the face and limbs were the most affected areas of the skin, both more frequent in confirmed CHIKV-infected patients. Some of the total of patients had mucosal ulcers (*n* = 14, 2.6%); however, the presence of such ulcers in the genital area was significantly associated with confirmed CHIKV-infected patients (Table 3).

**Table 3.** Dermatological involvement in patients with confirmed CHIKV infection.

	IgG or IgM for CHIKV		** **	
	*Positive*^a^** **(*n* = 295)	*Negative*^b^** **(*n* = 253)	OR (CI)	*p-*value
*Rash* (*n*: 151)				
Face (*n*: 106)	94 (88.7%)	12 (11.3%)	9.39 (5.0–17.62)	<0.001
Thorax (*n*: 92)	84 (91.3%)	8 (8.7%)	12.19 (5.76–25.76)	<0.001
Abdomen (*n*: 91)	84 (92.3%)	7 (7.7%)	13.99 (6.33–30.90)	<0.001
Back (*n*: 80)	73 (91.3%)	7 (8.7%)	11.55 (5.21–25.62.0)	<0.001
Limbs (*n*:105)	91 (86.7%)	14 (13.3%)	7.61 (4.20–13.77)	<0.001
*Pruritus* (*n*: 101)				
Presence of pruritus	87 (86.1%)	14 (13.9%)	7.14 (394–12.93)	<0.001
*Mucosal ulcers* (*n*: 14)				
Oral (*n*: 10)	9 (90.0%)	1 (10.0%)		
Genital (*n*: 13)	11 (84.6%)	2 (15.4%)	4.86 (1.06–22.14)	0.024

CHIKV: chikungunya virus; IgM: immunoglobulin M; IgG: immunoglobulin G; OR: odds ratio; CI: 95% confidence interval.

^a^fulfilled World Health Organization criteria for confirmed case of CHIKV; ^b^did not fulfilled World Health Organization criteria for confirmed case of CHIKV.

#### Gastrointestinal involvement

Gastrointestinal symptoms were the least common in the studied population (*n* = 103, 18.8%), with diarrhoea the most frequent in CHIKV-infected patients (Table 4).

**Table 4.** Gastrointestinal involvement in patients with confirmed CHIKV infection.

	IgG or IgM for CHIKV		** **	
	*Positive*^a^** **(*n* = 295)	*Negative*^b^** **(*n* = 253)	OR (CI)	*p-*value
*GI symptoms* (*n*: 103)				
Diarrhoea (*n*: 90)	75 (83.3%)	15 (16.7%)	5.40 (3.01–9.69)	<0.001
Emesis (*n*: 40)	33 (82.5%)	7 (17.5%)	4.42 (1.92–10.19)	<0.001
Nausea (*n*: 40)	34 (85.0%)	6 (15.0%)	5.36 (2.21–12.99)	<0.001
Abdominal pain (*n*: 30)	23 (76.7%)	7 (23.3%)	2.97 (1.25–7.04)	<0.01

CHIKV: chikungunya virus; IgM: immunoglobulin M; IgG: immunoglobulin G; OR: odds ratio; CI: 95% confidence interval; GI: gastrointestinal.

^a^fulfilled World Health Organization criteria for confirmed case of CHIKV; ^b^did not fulfilled World Health Organization criteria for confirmed case of CHIKV.

## Discussion

Our study showed that overall, half of the patients with suspected CHIKV infection had confirmatory serological tests. Low income (below 157 USD per month) and low socioeconomic stratum (stratum 1) were associated with CHIKV infection.

The variety of altitudes and ecosystems in Colombia allows for multiple mosquito species including *Ae. aegypti* and *Ae. albopictus*, facilitating the rapid spread of a disease like chikungunya. Because Colombia’s first epidemic of chikungunya coincided with a COPCORD study, we were able to characterize the cases of CHIKV infection with information from multiple regions of the country. Our data were obtained directly from patients, decreasing selection bias. Our study was performed from August 2014 to September 2015, and therefore, did not overlap with the Zika virus epidemic in Colombia that started in October 2015. Consequently, no serology for Zika virus was needed in our study group. Also, up to now, no autochthonous cases have been reported for the Mayaro virus [39].

The COPCORD study included 2336 surveys from Bogotá, 1220 each from Medellin and Cali, 746 from Barranquilla, and 503 each from Bucaramanga and Cúcuta [30]. As in other studies, the northern Caribbean region of Colombia, represented in our study by Barranquilla, was the most affected [34,40–42]. However, in contrast to previously published findings [40,43–46], the southwest (Cali) and northeast (Bucaramanga and Cúcuta) had fewer reported cases in our study. This could be because at the time of patient assessment in those cities, the CHIKV epidemic had just started (end of 2014), while the published findings from those regions are from the peak of the epidemic in early and mid-2015. Interestingly, many patients with suspected CHIKV were reported from cities like Bogotá or Medellin, where CHIKV and ZIKA are not endemic due to the absence of *Ae. aegypti*. One explanation is that although those patients resided in Bogotá or Medellin, their infection was acquired from surrounding municipalities where CHIKV was endemic [20,40].

Most studied patients were female, consistent with reports from Malaysia, Réunion, and the Comoro Islands, and other reports from Colombia [20,23,47–49]. A selection bias is present, which must be considered. Nevertheless, to date, it has not been demonstrated that the CHIKV or its vectors have a sex predilection. Indeed, a study in Mayotte found higher seroprevalence in males, suggesting that inconsistencies in sex preponderance are related to differences in exposure due to community-specific habits, customs, or behaviours [50].

Although most interviewed subjects had low socioeconomic status, which create a selection bias, this high percentage reflects the current state of the country. Our finding is consistent with the study in Mayotte where poor living conditions were associated with high risk of CHIKV infection[50]. A recent study conducted in Barranquilla showed that the CHIKV epidemic affected mostly the poorest communities [34]. Moreover, in a study from Cali the authors observed clustering of homicide rates, lower social strata, and increased risk of arboviral infections (CHIKV and dengue), supporting the hypothesis that reported violence impacts disease risk [43]. Another study in two neighbouring municipalities from the northern region of the country demonstrated that a high number of families with low income in one municipality was associated with high health vulnerability [41]. In other arboviral infections like dengue, studies have found associations between higher incidences of infection and lower socioeconomic status [51,52]. Unfortunately, our study did not evaluate other variables related to disease acquisition, which could increase the precision of our findings.

Consistent with other studies, we found symmetric arthralgia to be a frequent symptom in patients with suspected CHIKV infection [8,53,54], with knees, hands, and ankles being the most affected joints [8,53–56]. In particular, ankles, hand joints, feet joints, knees, and wrists were the sites with more symmetric arthritis in patients with confirmed CHIKV infection. These findings suggest that symmetric arthritis of these joints is characteristic of CHIKV infection and should be key in diagnosing it.

Fever was a frequent symptom in our CHIKV-infected patients. Other studies have found similarly high rates of fever ranging from 90 to 100%; however, it is an obligatory symptom for WHO CHIKV infection criteria, increasing selection bias [47,54–59]. Another point to consider is that in regions where Zika, dengue, or CHIKV infections co-exist simultaneously, the use of non-specific symptoms like fever in the diagnostic criteria increases sensitivity but decreases specificity, reducing the ability to discern which infection is responsible; this can lead to over- or underdiagnosis. An example of the use of more specific symptoms in the diagnosis of an arboviral disease was demonstrated by Braga et al. They found that using the presence of rash, pruritus, and conjunctival hyperaemia, and excluding fever, anorexia, and petechiae increased performance when compared to other existing Zika suspected case definitions [60]. The same could be true with other systemic symptoms like fatigue and myalgia (found frequently in our cohort). These are present in high percentages in CHIKV infection descriptions but can also be found in other arboviral infections [61–63].

Our study showed a higher frequency of maculopapular rash in face and limbs with pruritus in almost half of the patients. Other studies in CHIKV epidemics have shown similar findings [54,64]. However, Zika also presents with maculopapular rash [61]. Indeed, Braga et al used maculopapular rash and pruritus as one of the hallmarks of their disease case definition [60]. Our patients did not report conjunctivitis and we found an important percentage of mucosal ulcers in CHIKV-confirmed patients; these are less frequent in Zika and dengue infections. Other studies have shown the presence of mucosal ulcers in CHIKV infection. However, this sign is not frequently evaluated because it is used mainly in studies with a dermatological focus [65]. More emphasis should be placed on the clinical examination of mucosal ulcers in CHIKV-suspected patients, because these symptoms could play an important role in differentiating CHIKV from Zika or dengue infections.

Almost half the patients with confirmed CHIKV infection suffered from gastrointestinal symptoms, especially diarrhoea. Other studies have found similar frequencies of gastrointestinal involvement; however, these symptoms are not included in the case definition of CHIKV infection [54–57,59]. One explanation may be that gastrointestinal symptoms are also frequent in other arboviral infections, nevertheless, they are far more frequent in CHIKV infection [63,66].

Our study has some limitations. First, there is a selection bias regarding MSK symptoms since the study was developed within the framework of a COPCORD methodology. Another selection bias is related to the subjects evaluated in a house-to-house system, housewives in our case. Further, the fact that the patients were evaluated in their homes and not a medical setting decreased our chances of capturing more severe cases. However, it did allow us to detect asymptomatic patients who otherwise would not have consulted a physician. Second, there is recall bias of the symptoms that could not be corroborated by the medical evaluator. Third, there was no confirmation of the disease by real-time polymerase chain reaction (RT–PCR) due to high costs. However, because this was the first chikungunya epidemic in Colombia, it is assumed that the population is immunologically naïve. Furthermore, because some patients were evaluated after infection, we were not able to test viral loads, which would have been helpful to create a timeline of the infection. Finally, we did not perform dengue serology to evaluate cross-reactions with CHIKV. However, the manufacturer of the ELISA kits ensures no cross-reactivity, as well as specificity and sensitivity >90% for both IgG and IgM.

Our study also has some strengths. Because it was developed within the framework of a COPCORD methodology, a great amount of data could be gathered. Colombia’s population is also reasonably represented by the study, considering the number of evaluated patients in six geographically diverse cities. Another strength is that all patients were evaluated by a rheumatologist or a rheumatology fellow ensuring the accuracy of physical examination, especially the MKX examination.

Our study showed that low socioeconomic status and low income are associated with CHIKV infection. Public health strategies on prevention, education, and vector control should prioritize vulnerable communities as well as decreasing health inequalities and social disparities.

A considerable number of patients do not display the “typical or classical” symptoms of CHIKV infection, leading to underreporting and underdiagnosis. A distinctive clinical picture is presented by CHIKV infection with symmetrical arthritis of hand joints, ankles, and feet joints as the hallmark for diagnostic clinical criteria. Although fever, myalgia, and fatigue are present in high percentages in CHIKV-confirmed patients, Zika and dengue infections can also produce these symptoms, which decreases their usefulness for clinical diagnosis in a primary care setting. More distinctive clinical features of each disease are more useful for the primary care physician to diagnose arboviral diseases.

Further studies are needed to investigate why some patients display fever and other do not, as well as why MSK symptoms are more severe or chronic in some patients than in others. We believe that our study is a good foundation for future research into these additional questions.
